# The Truth about Medical Record Cards

**Published:** 1921-03-05

**Authors:** 


					The Truth About Medical Record Cards.
There has been a noteworthy slump in the news-
paper criticisms of the new system of medical cards
for insured persons since the Ministry of Health
issued a terse statement in which are set forth,
without comment, the plain facts showing precisely
how the scheme has been received by those whom
it most directly concerns. As our readers are
aware, we have all along expressed the opinion that
the state of public feeling in this matter has been
absurdly exaggerated in the press, and it is now
perfectly clear that the indignation which was sup-
posed to be moving doctors and insured persons to
revolt in all parts of the country existed only in the
imagination of a number of editors eager for a pos-
sible chance of casting discredit upon a State
ago sing,ed out iot *
Out of 10C000n?nV officJally. announced are these-
five have 'marl ' ')10ve<^ SOcieties and branches on y
panel conri^ff 8 to Dr' Addis?' ?n(1
sentations hav ^ S same number of I'ep1?,
representatinn?6 e1majlafce^- These proposals an.
detail as fo ? 1 rfate7- in th* main to points <>
adinittedlv rW 1Cji a ^us'men^s are in some case
templation and are' we believe, in ^
minister r i U1,ance committees, which a,
oountv- bBDefit in W Tr
Tn fact onA ? *' .Ve mafJe 110 complaint whaJ?v :
? resolntZ rWnmittee' by 21 vote* to 2> r6] te
disapproving- of the cards. Inaccura
March 5, 1921, THE HOSPITAL.
519
THE PUBLIC WELL-BEING?(continued).
?statements have been published concerning the cost
?f the new cards. They will actually cost less than
the original cards, and will possess the additional
advantage of forming a permanent record. The
cost to insurance committees of filling in and send-
lng the cards to practitioners in England and Wales
^ill, it is estimated, not exceed ?1.900.
The one point of practical importance which we
hope will be considered by the Government is the
^ability of these personal records to be disclosed in
ligation in the courts. It is no answer to say
that cases in which such a question may arise will
' Q comparatively rare. The liability is one that
?ught not to exist, and the only sure safeguard is to
Jl}ake all such documents " privileged." Discus-
sion of this matter may again raise the whole ques-
??n in its broadest aspect of the doctor's obligations
in regard to secrecy. The best course is to trust
the doctor. EVery British medical man who is
worth his salt knows well enough how to interpret
the general rule of guiding his conduct; and in
those special individual cases in which the honour
or safety or innocence of a third person is involved,
| the doctor must follow the dictates of his conscience
according to the merits of a particular case, and act
J upon his judgment in the light of his knowledge of
all the surrounding circumstances and the character
0'f the implications. A question bearing on this
point was asked, in the House of Commons a few
days ago, but Dr. Addison, in a written answer,
! did not commit himself to any definite proposals,
I though he expressed an opinion, for what it may
j be legally worth, that no action would lie against
a medical man compelled to make damaging dis-
closures in respect of the medical cards.

				

